# Magnetotransport and magnetic properties of amorphous $\mathrm{NdNi}_5$ thin films

**DOI:** 10.1038/s41598-020-70646-2

**Published:** 2020-08-13

**Authors:** Carla Cirillo, Carlo Barone, Harry Bradshaw, Francesca Urban, Angelo Di Bernardo, Costantino Mauro, Jason W. A. Robinson, Sergio Pagano, Carmine Attanasio

**Affiliations:** 1 CNR-SPIN, c/o Università degli Studi di Salerno, 84084 Fisciano, SA Italy; 2 Dipartimento di Fisica “E. R. Caianiello”, Università degli Studi di Salerno, 84084 Fisciano, SA Italy; 3 INFN Gruppo Collegato di Salerno, c/o Università degli Studi di Salerno, 84084 Fisciano, SA Italy; 4 Department of Materials Science & Metallurgy, University of Cambridge, 27 Charles Babbage Road, Cambridge, CB3 0FS UK; 5 Fachbereich Physik, Universität Konstanz, Universitätsstraße 10, 78464 Konstanz, Germany

**Keywords:** Condensed-matter physics, Materials for devices

## Abstract

$\mathrm{NdNi}_5$ is an intermetallic compound with a bulk Curie temperature ($T_{\mathrm{Curie}}$) of 6–13 K. While existing studies have focused on $\mathrm{NdNi}_5$ crystals, amorphous thin-films of $\mathrm{NdNi}_5$ are potentially important since they would be magnetically soft without magnetocrystalline anisotropy, meaning that small external magnetic fields could reverse the direction of their magnetization. Here, we report $\mathrm{NdNi}_5$ thin-films with a thickness in the 5–200 nm range, deposited by DC magnetron sputtering onto Si(100). Films are amorphous with a weak temperature-dependent resistivity with values ranging between 150 and 300 $\upmu \Omega$ cm. By means of noise spectroscopy, by analyzing the time-dependence of fluctuation-induced voltages, it is found that at low temperatures the resistance fluctuations are due to the Kondo effect. Volume magnetometry indicates $T_{\mathrm{Curie}} = 70$ K with a magnetic coercive field of 30 mT at 5 K for a 125-nm-thick film. The results are promising for the development of Ferromagnet(F)/Superconductor(S)/Ferromagnet(F) pseudo spin-valve devices based on amorphous $\mathrm{NdNi}_5$ thin films.

## Introduction

Spintronics is a major field of research in condensed matter physics^1^. The capability to create and manipulate electronic spin currents enables to realize spin-based devices which, compared to their traditional charge-based counterpart, can be faster and demand lower power^1^. The birth of spintronics traces back to the discovery of giant magnetoresistance (GMR) in Fe/Cr synthetic antiferromagnetic multilayers in which the electrical resistance increases when the magnetization in the magnetic layers changes from a parallel to an antiparallel alignment^2,3^. A large number of material systems have been proposed for spintronics^4^ including superconductors in conjunction with magnetic materials. This has paved the way for superconducting spintronics, which has potential to lead to the development of circuits that are more energy efficient, meaning that they should generate less heat and require low power for their functioning^5,6^. In particular, a ferromagnet with a small value of the coercive field, $H_{\mathrm{c}}$, can be easily tuned in its magnetic properties, going from a state in which the magnetization, *M*, is zero when the applied external magnetic field *H* is equal to $H_{\mathrm{c}}$ to a state in which, for $H > H_{\mathrm{c}}$, $M \ne 0$. In superconducting/ferromagnetic (S/F) systems, this property can push the S/F system from the normal to superconducting state at low temperatures. Such a system can be used as a superconducting valve, since it can be switched between a superconducting (ON) and a normal (OFF) state by controlling the value of *H*.

$\mathrm{NdNi}_5$ is an intermetallic compound belonging to the $\hbox {RENi}_5$ (RE = Rare-earth) series. In bulk form, it is characterized by a large magnetocrystalline anisotropy, with the easy (hard) axis lying along the *a* axis within the hexagonal plane (the *c* axis) and an *a* axis (*c* axis) magnetization at 35 T of 3.3 *μ*_B_/f.u. (1.65 *μ*_B_/f.u.)^7,8^, and a $T_{\text{Curie}}$ in the range of 6–13 K^8–11^. Here *μ*_B_ is the Bohr magneton. In order to be used in conjunction with superconductors such as Nb ($T_{\mathrm{c}} \sim 9$ K, *T*_c_ is the superconducting transition temperature) or NbN ($T_{\mathrm{c}} \sim 15$ K), the most frequently used materials in superconducting electronics, it is crucial to realize good quality $\mathrm{NdNi}_5$ thin films because their availability may enable the design of dedicated transport experiments based on heterostructures operating as S/F-based devices. However, currently $\mathrm{NdNi}_5$ is only available in crystal form^8,11,12^ and, to the best of our knowledge, thin films have not been fabricated. Furthermore, it would be useful to obtain non-crystalline $\mathrm{NdNi}_5$ films since amorphous magnetic materials are typically characterized by narrow hysteresis loops. This makes possible to use very small external magnetic fields to change their magnetic properties and in particular to reverse the direction of magnetization^13^. Ferromagnetic thin films with small values of $H_{\mathrm{c}}$ can also be used in electronic applications where small intensity local fields are available to record the logic state, as in the case of magnetic superconducting memories, both Josephson junctions^14,15^ and nanowires-based devices^16^. Moreover, due to their disordered structure, amorphous materials are robust against the presence of impurities and then, in general, easier to fabricate, which is another important property for their potential applications. These are some of the reasons why the study of this class of materials has recently encountered an increasing interest in spintronics and other research fields related to it^17^.

## Results

### Structural properties

**Figure 1:**
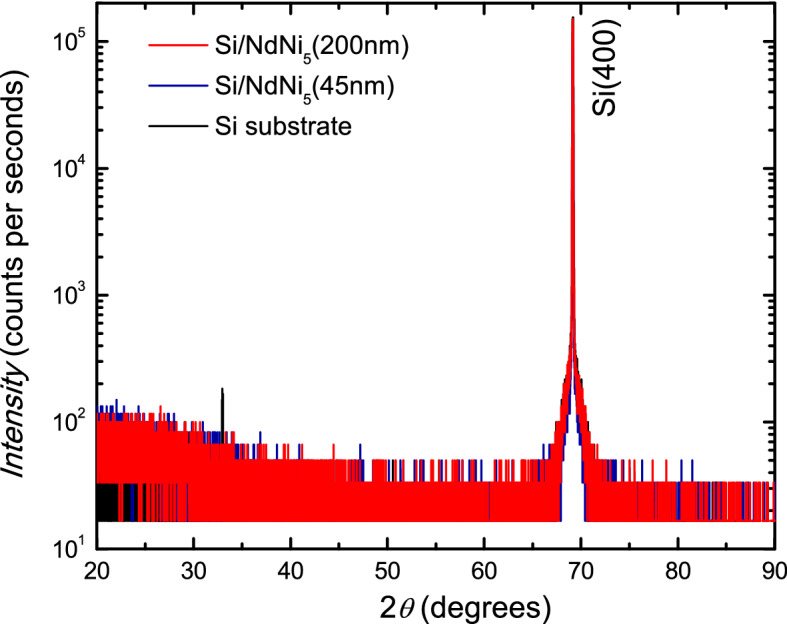
Structural characterization. High-angle X-ray diffraction traces from a 200-nm- (red curve) and a 45-nm-thick (blue curve) $\mathrm{NdNi}_{5}$ films on Si(100). The black curve is the trace from the bare substrate.

$\mathrm{NdNi}_{5}$ crystallizes in a $\hbox {CaCu}_5$-type hexagonal structure (space group *P*6/*mmm*)^10^ with lattice parameters $a=4.952 \, {\AA }$ and $c=3.976 \, {\AA }$^8,10^. X-ray diffraction traces on polycrystalline $\mathrm{NdNi}_{5}$ show their highest-intensity peaks at $2\theta$ = $31^\circ$ for the $\mathrm{NdNi}_5$ (101) diffraction and at $2\theta$ = $42^\circ$ for the $\mathrm{NdNi}_5$ (111) diffraction^8^. The thin films investigated in this study are amorphous, as determined through high resolution X-ray diffraction (XRD) measurements performed in grazing incident configuration. Figure 1 shows high-angle XRD data of 45- and 200-nm-thick $\mathrm{NdNi}_{5}$ films on Si(100). For both films, the only peak detected is the one related to the (400) diffraction planes of Si. The small-intensity peak observed at $2\theta = 33^\circ$ in the spectrum of the bare substrate is due to the basis-forbidden reflection from the Si(200) planes^18, 19^. As reported previously, it may sometimes occur due to multiple diffractions which can be present when X-ray patterns are acquired using a $\omega -2\theta$ scan^19^. In supplementary Fig.S1 we show an additional spectrum taken with a different diffractometer on a 200-nm-thick $\mathrm{NdNi}_{5}$ film deposited on a different substrate, namely $\mathrm Al_2O_3 (1120)$. Again, only diffraction peaks connected to the substrate are present.

### Transport properties

The resistivity, $\rho$, of the amorphous $\mathrm{NdNi}_{5}$ (from now on called *a*-$\mathrm{NdNi}_5$) thin films is measured from room temperature down to $T=4.2$ K, using a van der Pauw configuration^20^. Previous studies, on single crystals or polycrystalline $\mathrm{NdNi}_{5}$ samples^8,11^, show pronounced anomalies in the resistivity at $T \sim 7.2$ K, close to the magnetic ordering temperature^10^. However, differences in the transport properties originate from the loss of periodic arrangement. While for a crystal $\rho$ is related to electron scattering processes with defects and oscillating ions, the absence of a periodic potential in an amorphous solid generates a diffusive electronic regime, governed by a mean free path, $\ell$, of the order of the interatomic distances^21^. In particular, in the case of magnetic amorphous alloys $\rho$ can be strongly affected by magnetic ordering and Kondo effect and can increase below the ordering temperature^22^. At the moment, no information on the transport properties of *a*-$\mathrm{NdNi}_5$ thin films are available in the literature. However, the amorphousness should determine a dramatic change of the transport properties with respect to the crystalline samples. In particular, according to the Mooij criterion^23^, the temperature coefficient of the resistivity $\alpha = (1/\rho ) (d\rho /dT)$ of disordered and amorphous metallic systems and alloys containing transition metals is predicted to be almost temperature independent. Furthermore, the decrease in the slope of $\rho$ as a function of *T* is related to the increase of $\rho$. For values of $\rho \sim$ 100–200 $\upmu \Omega$ cm, $\alpha$ is almost zero. Also negative values of $\alpha$ are observed. However, in the case of magnetic systems this happens in a small range of *T*, of the order of few Kelvin, close to the magnetic transition^8,11,23^. In the case of magnetic amorphous alloys this behavior, studied by means of electron-transport measurements, is attributed to weak-localization (WL) effects^24–26^ rather than to Kondo mechanism, which is related to the presence of localized magnetic impurities in the system^27^.

**Figure 2:**
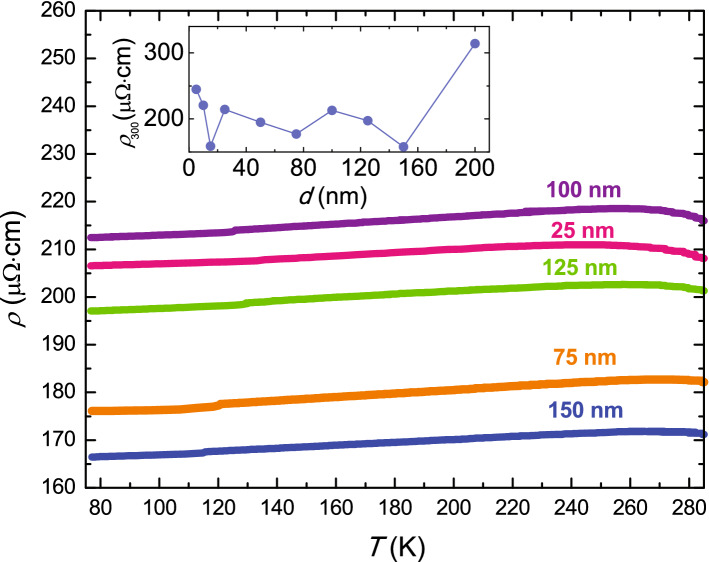
Resistivity results. $\rho$ versus temperature of *a*-$\mathrm{NdNi}_5$ films with thicknesses ranging from 25 nm to 150 nm. Inset: $\rho _{300}$ as a function of the thickness of *a*-$\mathrm{NdNi}_5$ films.

Figure 2 shows the temperature-dependence of the resistivity of *a*-$$\mathrm{NdNi}_5$$ films of different thickness. The values of $$\rho$$ approximately range from 160 to $$300\,\upmu \Omega$$ cm and they are weakly-dependent on *T*. These values are similar to those observed in magnetic amorphous alloys containing rare earth elements^22^. The high $$\rho$$ of the amorphous samples is mainly due to lack of structural order, indicating a small value of $$\ell$$. A rough estimate of $$\ell$$ is made on the basis of the Drude model of conduction in metals even if it is not strictly applicable for amorphous systems since in this case the electronic transport is diffusive and not ballistic, meaning that electron-phonon interaction plays a relevant role^28^. Furthermore, in magnetic metallic glasses containing rare earth elements, the resistivity below $$T_{\mathrm{Curie}}$$ is further increased due to the fact that the spin correlation function is spatially coherent only over very short distances^21,22^. In the Drude expression of the mean free path, $$\ell = (v_{\mathrm{F}} m_e)/(n_ee^2\rho )$$, the Fermi velocity, $$v_{\mathrm{F}}$$, and the value of the electronic densities at the Fermi level can be estimated as an average of the corresponding quantities of the single elements weighted by their atomic percentage ($$n_e^{\text{Nd}} = 5.60 \cdot 10^{22}\,{\text{cm}}^{-3}$$, $$n_e^{\text{Ni}} = 1.82 \cdot 10^{23}\,{\text{cm}}^{-3}$$, $$v_{\text{F}}^{\mathrm{Nd}} = 1.7 \cdot 10^8\,{\text{cm/s}}$$, $$v_{\text{F}}^{\mathrm{Ni}} = 3.2 \cdot 10^8 \,{\text{cm/s}})$$^29^. Therefore, using $$\rho =230\,\upmu \Omega$$ cm we estimate $$\ell \sim 0.3$$ nm, a value which is smaller than the interatomic distances of crystalline $$\mathrm{NdNi}_5$$. This result indicates that, according to the Ioffe-Regel criterion^30^, the nearly free electron theory of electrical conduction in metals is a poor approximation and other scattering mechanisms should be invoked to describe the conduction in our amorphous films. In this respect, a more detailed analysis is needed to better clarify this point. The slope of the linear part of the curves (approximately from $$T \sim 280$$ K down to $$T \sim 80$$ K) is evaluated for all the samples. The calculated values of $$\alpha$$ are all extremely small, almost temperature independent (at $$T = 80$$ K we find $$\alpha \sim 2 \times 10^{-5}$$$$\hbox {K}^{-1}$$ for the 125-nm-thick film) and even slightly negative for larger values of $$\rho$$, in reasonable agreement with the Mooij criterion^23^.

In the inset of Fig. 2 we have plotted the resistivity at room temperature, $\rho _{300}$, as a function of the thickness, *d*, of the *a*-$\mathrm{NdNi}_5$ films. $\rho _{300}$ does not show any definite trend over the entire thickness range investigated, as expected for amorphous films. Due to the high resistivity of the samples (one order of magnitude larger than for polycrystals of $\mathrm{NdNi}_5$)^11^ related to the absence of lattice ordering, the contribution to the resistivity connected to the scattering of electrons at the interfaces of the film with both the substrate and the vacuum is negligible. For this reason, the Fuchs-Sondheimer behavior^31^ which, in the case of thin metallic films, predicts an increase of $\rho$ when *d* is reduced, it is not reproduced.

**Figure 3:**
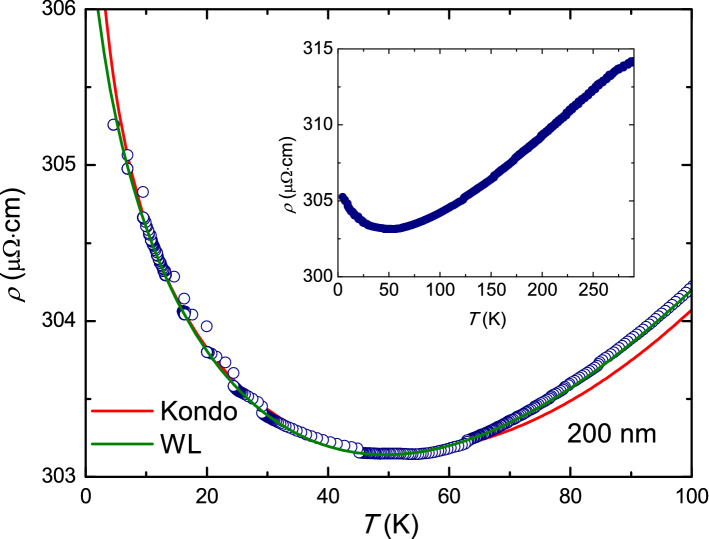
Upturn of the resistivity at low temperatures. Low-temperature behavior of the resistivity of the 200-nm-thick *a*-$\mathrm{NdNi}_5$ film. The red (green) line is the fit to the data with the Kondo (weak-localization) model, see text for details. The inset shows the curve over the full measured temperature range.

Figure 3 shows on an enlarged scale the low-temperature part of the $$\rho (T)$$ curve of the 200-nm-thick film, chosen as a representative sample (in the inset we show the $$\rho (T)$$ curve over the full temperature range). The slope of the linear part of the curve (from $$T \sim 280$$ K down to $$T \sim 80$$ K) is evaluated and at $$T = 170$$ K it is $$\alpha \sim 10^{-4}$$$$\hbox {K}^{-1}$$. The presence of a minimum and an upturn in the temperature dependence of the resistivity is analyzed in terms of a Kondo-like mechanism or a strong electron-electron (e-e) interaction. The data are fitted using both models, as shown in Fig. 3. Kondo model and e-e interaction describe different physical mechanisms. The former relates the modification of resistivity to the electron scattering with magnetic impurities and gives a logarithmic type dependence of $$\rho$$ on the temperature^27^$$\rho \sim -A\,lnT + B\,T^n$$, where the coefficient *A* is the so-called Kondo term. To reproduce the minimum in the $$\rho$$ versus *T* curve, *A* has to be positive. On the other hand, in highly inhomogeneous samples, where weak-localization phenomena are the main factor affecting the transport properties, e-e interactions are the predominant mechanism in determining the conduction of the system^32^. In this case $$\rho (T) \sim C/\sqrt{T} + D\,T^n$$. The exponent *n* in both models is equal to 2, when the temperature dependence of the resistivity is due to e-e scattering, or to 5 in the case of lattice resistance^28^. In the case of inhomogeneous and disordered material different values of *n* are possible. In the case of granular aluminum oxide films the value $$n=1$$ has been obtained^33,34^. The experimental data are reproduced for both the models with good accuracy, see green and red lines in Fig. 3, and realistic fitting parameters ($$A=0.63 \pm 0.03 \, \upmu \Omega$$ cm, $$B=0.046 \pm 0.002 \, \upmu \Omega \,\hbox {cm} \hbox {K}^{-1}$$ and $$n=1.02 \pm 0.03$$ for the Kondo model, $$C=12.1 \pm 0.6\, \upmu \Omega \cdot \,\hbox {cm} \, \hbox {K}^{1/2}$$, $$D=0.046 \pm 0.002 \, \upmu \Omega \,\hbox {cm} \, \hbox {K}^{-1}$$, and $$n=1.03 \pm 0.05$$ in the case of weak-localization). Moreover, the statistical parameters associated to the fitting to the experimental data are very similar. In particular, the reduced $$\chi ^2$$ is equal to $$5.2 \times 10^{-3}$$ for the Kondo model and to $$14.7 \times 10^{-3}$$ in the case of weak-localization. The coefficient of correlation is $$r^2=0.998$$ for both the models. This makes impossible, based only on the electric transport characterization, to identify the physical mechanism responsible of the upturn in the temperature dependence of the resistivity in our samples. As we will show in detail later on, noise spectroscopy measurements help to shed light on this important issue.

### Magnetic properties

**Figure 4:**
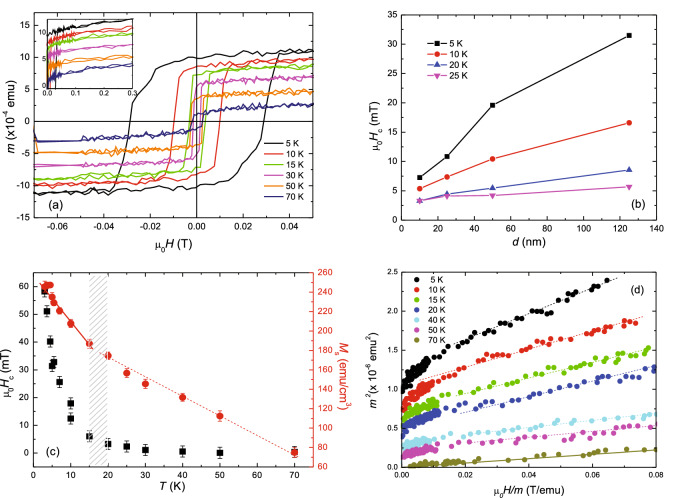
Magnetic characterization. (**a**) Low-field magnetization loops for the *a*-$\mathrm{NdNi}_5$ 125-nm-thick film between 5 and 70 K. The inset shows curves obtained for positive high fields. (**b**) Coercive field ($H_{\mathrm{c}}$) as a function of *a*-$\mathrm{NdNi}_5$ film thickness at four different temperatures. (**c**) Temperature dependence of $H_{\mathrm{c}}$ (left axis) and magnetization at 1 T, $M_{\mathrm{s}}$ (right axis), for the *a*-$\mathrm{NdNi}_5$ 125-nm-thick film. The shadowed area indicates the temperature range where a change in the slope of both $H_{\mathrm{c}}$ and $M_{\mathrm{s}}$ is observed. (**d**) Arrot plots obtained from the *m*(*H*) data measured at different temperatures for the 125-nm-thick film. The solid line is the fit to the high-field data measured at $T=70$ K. The dotted lines are guides to the eye.

Magnetization loops are measured at different temperatures on several samples of different thickness, as reported for example in Fig. 4a where the magnetic moment, *m*(*H*), at low fields is shown for the 125-nm-thick film. In the inset of the same figure, the high-field magnetization data at the same representative temperatures are presented. It is evident that the magnetic moment does not show a full saturation up to 1.0 T. This result confirms what already measured on polycrystalline samples where this effect is connected to the magnetic anisotropy of the material^7,8,11^. The low-field data allow to estimate the value of the coercive field, $H_{\mathrm{c}}$, at different temperatures. In Fig. 4b $H_{\mathrm{c}}$ is plotted as a function of the thickness of the films at four different temperatures. For larger *d* all the curves show an increase of the values of the coercive field as well as a stronger temperature dependence. Due to the amorphousness of the samples, the values of $H_{\mathrm{c}}$ are very small, namely, for the thicker measured film, $H_{\mathrm{c}}$ is of the order of 30 mT at $\mathrm T=5$ K. The temperature dependence of both $H_{\mathrm{c}}$ and the value of the magnetization at 1 T, $M_{\mathrm{s}}$, for the 125-nm-thick film are summarized in Fig. 4c. At $T \sim 20$ K both $H_{\mathrm{c}}$ and $M_{\mathrm{s}}(T)$ show a change in the slope but they remain significantly different from zero up to 70 K. The positive inflexion in $M_{\mathrm{s}}$ suggests an additional moment which can potentially come from nanoscale magnetic clusters having a low $T_{\mathrm{Curie}}$. This is consistent with the rise in $H_{\mathrm{c}}$ below $T=20$ K, as the onset of magnetic particles is likely to enhance pinning of domain walls, as reported also for amorphous films^35^. Even not clearly visible, due to a small remanent moment trapped in the superconducting magnet, the value of *H*_c_ turns out to be negative for $T>25$ K. The low-temperature $M_{\mathrm{s}}$ value of $\sim 0.3 \, \mu _{\mathrm{B}}/\mathrm{atom}$, measured at $T=5$ K, is almost one third smaller then those reported in the literature for polycrystalline samples^7,8,11^ and it does not significantly depend on *d*. To further demonstrate the appearance of the ferromagnetism in the thin film, in Fig. 4d we plot $m^2$ as a function of *H*/*m* for different temperatures, the so-called Arrot plots^36^. The slope of the curves is positive for all the temperatures which indicates a second-order ferromagnetic phase transition. Moreover, from the extrapolation of the curves to $H/m=0$ it results that for $T=70$ K the line intercepts the origin of the axes. This value of temperature is then identified as $T_{\mathrm{Curie}}$^37,38^.

### Electric noise spectroscopy

**Figure 5:**
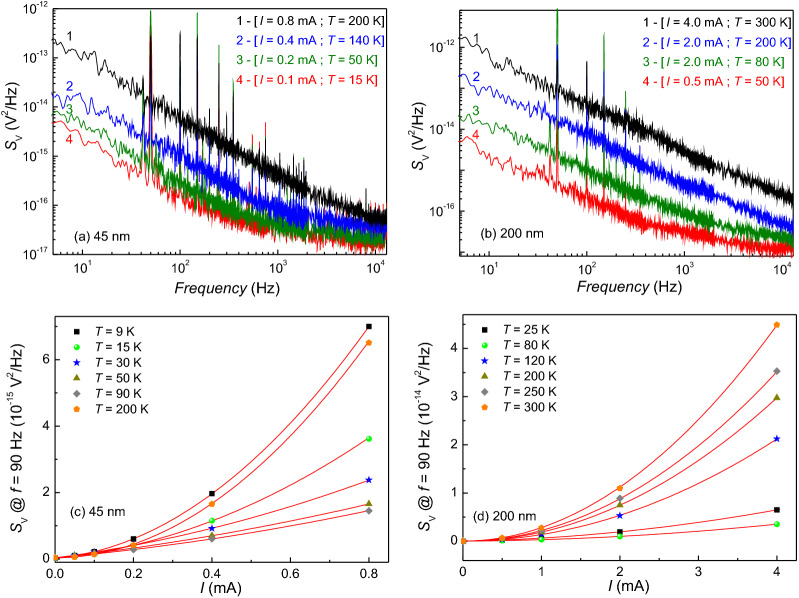
Voltage-noise experimental behaviour. (**a**, **b**) Voltage-noise spectra of the 45 nm (200 nm) thick film at different temperatures and bias currents. (**c**, **d**) The current dependence of the 1/f noise amplitude in the temperature range 9-200 K (25–300 K) for the 45-(200-)nm-thick sample.

The direct-current (DC) transport measurements show evidence for the existence of scattering mechanisms occurring at temperatures below 70 K, where a sign of magnetic activity is observed. In this low-temperature regime, the possible coexistence of electron-electron type interaction and magnetic Kondo effect cannot be excluded. Moreover, in determining the electrical transport of *a*-$\mathrm{NdNi}_5$ thin films, the magnetic behavior observed near 20 K could also play a role. A powerful experimental technique, which is able to evidence subtle effects on charge motion, is given by noise spectroscopy, which analyzes the time dependence of fluctuation-induced voltages. Noise spectroscopy gives a detailed insight into the basic electrical conduction mechanisms and can be a very informative methodology to understand the kinetic processes and the dynamic behaviors of the charge carriers in the investigated systems, as already demonstrated in a large variety of materials^39^ and devices^40,41^. For all the investigated samples, having different thickness [45 nm, Fig. 5a and 200 nm, Fig. 5b] the voltage-spectral density $S_{\mathrm{V}}$ shows two different frequency (f) components in the whole temperature range: a 1/f-type noise in the low-frequency region and a “white”  frequency-independent noise at higher frequencies.

**Figure 6:**
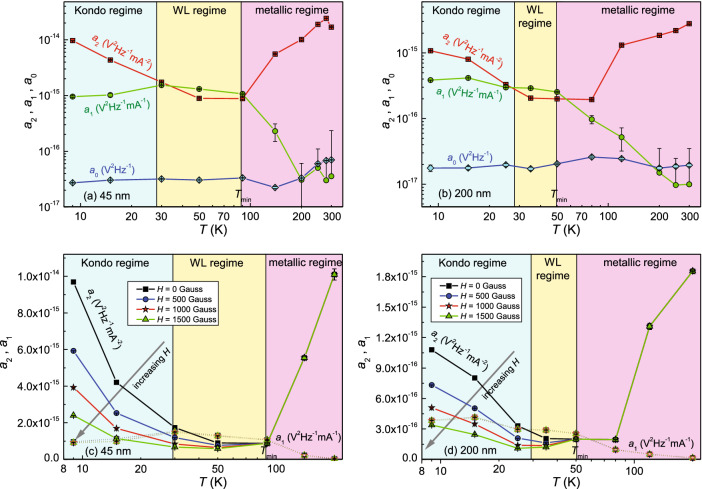
Temperature dependence of the noise parameters. (**a**, **b**) Temperature dependence of the noise coefficients $a_{2}$, $a_{1}$ and $a_{0}$ of Equation 1 for the 45-(200-)nm-thick sample. (**c**, **d**) The magnetic behavior of the noise coefficients $a_{2}$ and $a_{1}$ is shown in the temperature range 8-220 K for the 45-(200-)nm-thick film.

More information on the fluctuation mechanisms and, consequently, on the electric transport can be extracted by studying the bias current dependencies of the noise amplitude components. In particular: (I) resistance fluctuations are usually characterized by a quadratic current dependence of the 1/f noise^39^, (II) nonequilibrium universal conductance fluctuations produce a linear bias dependence of the 1/f noise^42,43^, (III) fluctuation-induced tunneling processes show an unusual quadratic current dependence of the “white” noise^44^. In the case of our samples, this last mechanism is ruled out, as the frequency-independent part of the experimental does not depend on the current bias and therefore is attributed to the thermal Johnson noise added to the instrumental background noise. In order to investigate the other two fluctuation processes, an analysis of the 1/f component is done as a function of the current and is shown in Fig. 5c, d at temperatures between 9 and 300 K. The noise data, shown at the reference frequency of 90 Hz, arbitrarily chosen in the spectral region where the spectrum background is free from external disturbances, can be reproduced in terms of a generic quadratic polynomial

1 $$\begin{aligned} S_{V}\left( 90~Hz\right) =a_{2}\left( T\right) I^{2}+a_{1}\left( T\right) I+a_{0}\left( T\right) \end{aligned}$$

where the constant term $a_{0}$ represents the value of the bias-independent “white” noise amplitude. The good agreement of the simple expression of Equation 1 to the data is testified by the red solid lines in Fig. 5c, d. This makes possible a precise evaluation of the noise parameters $a_{2}$ and $a_{1}$, whose temperature dependence is shown in Fig. 6.

The most evident fingerprint of Fig. 6 is the very similar behavior of $a_{2}$ and $a_{1}$ coefficients, both for the thinner film [(panel (a)] and for the thicker one [(panel (b)]. It can be also observed that, above the temperature where the resistivity has the minimum, $T_{\mathrm{min}}$, the quadratic current coefficient $a_{2}$ is dominant. Therefore, resistance fluctuations are the noise source, as usually observed in a metallic regime in many materials. Between $T_{\mathrm{min}}$ and $T \sim 27$ K, the linear current coefficient $a_{1}$ becomes significant, indicating that in this case non-equilibrium universal conductance fluctuations are the noise source, as usually observed in a weak-localization regime. Below $T \sim 27$ K, the quadratic current coefficient $a_{2}$ increases, indicating that resistance fluctuations become relevant again although the $a_{1}$ parameter does not vanish, as in the metallic regime. Here, it is important to note that being $a_{2}$ and $a_{1}$ the quadratic and the linear current term, they affect differently the amplitude of the noise spectral density, depending on the value of the bias current.

The nature of these observed fluctuation processes can be understood by studying the magnetic field dependence of the noise components, as shown in Fig. 6, panels (c) and (d). These plots indicate that at high temperatures, above and around $T_{\mathrm{min}}$, no magnetic effect is visible. This is expected, for the applied values of the magnetic field (0–0.15 T), in the case of standard resistance fluctuations, characteristic of a metallic regime, and in the case of nonequilibrium universal conductance fluctuations, characteristic of a weak-localization regime. In the low-temperature region below 27 K, instead, the effect of a relatively weak magnetic field on the resistance fluctuations is clearly observed. The type of fluctuation detected cannot be attributed to the growth of ferromagnetic domains which is typically associated with a Barkhausen magnetic noise, whose frequency dependence is completely different from the observed one^45–47^. Moreover, as evident in Fig. 6, a reduction of the 1/f noise amplitude (i.e., $a_{2}$ coefficient) is measured by increasing the value of the applied magnetic field, and this effect is enhanced by lowering the temperature. This experimental finding, reported also for granular aluminum oxide thin films^33,34^, gives a further indication in favour of the occurrence of magnetic noise fluctuations to be ascribed to the presence of magnetic impurities found in a Kondo-like regime, as also observed in disordered amorphous alloys^22^.

## Discussion

We investigate amorphous $\mathrm{NdNi}_5$ thin films deposited by DC magnetron sputtering. The thickness of *a*-$\mathrm{NdNi}_5$ is varied between 5 and 200 nm with a resistivity of the order 250 $\upmu \Omega \,\hbox {cm}$, which is weakly temperature dependent. The upturn observed in the DC resistivity at low temperatures is interpreted as being due to a Kondo-like mechanism or strong electron-electron interactions, is analyzed using electronic noise spectroscopy. We find for temperatures below 100 K a linear current dependence of the 1/*f* noise amplitude, which is indicative of non-equilibrium universal conductance fluctuations characteristic of a weak-localization regime. For $T<25$ K Kondo effect contributes to the resistance fluctuation processes. The presence of these two different temperature regimes is also observed when measuring the temperature dependence of the magnetic moment of these samples which indicate a $T_{\mathrm{Curie}} = 70$ K in the 125-nm-thick sample. Interestingly, this study reveals the coexistence of Kondo effect and ferromagnetic ordering as already reported in different classes of crystalline materials, such as for instance ternary intermetallic Ce- and Np-based compounds^37,48,49^. Although unusual, this occurrence is not unexpected in amorphous ferromagnets, where structural and compositional disorder may produce a distribution of internal effective exchange field^50,51^, which could result in the increase in $T_{\mathrm{Curie}}$ observed in our amorphous films with respect to crystalline specimens^13^. Further experimental investigation, such as specific heat and magnetoresistance measurements as well as a deeper structural characterization, may help in gaining insight into this system^37,52^. Finally, it is important to underline that the values obtained for $H_{\mathrm{c}}$ and $M_{\mathrm{s}}$ are smaller than those for bulk and polycrystalline samples, and are very promising for the development of superconducting spin valves based on *a*-$\mathrm{NdNi}_5$ films.

## Methods

Thin films of *a*-$\mathrm{NdNi}_5$ in the thickness range of 5–200 nm are deposited in an ultra-high vacuum DC diode magnetron sputtering system on Si(100) substrates. Control films are sputtered on $\mathrm Al_2O_{3}(1120)$ to investigate the role of the substrate on the growth mechanism. By depositing the samples in different conditions, including the Ar pressure, $P_{\mathrm{Ar}}$, in the $10^{-3}$ mbar range, the substrate temperature, $T_{\mathrm{Sub}}$, and the sputtering power, *P*, no substantial differences are detected. The films investigated are grown at $P_{\mathrm{Ar}}= 6.4 \cdot 10^{-3}$ mbar, $T_{\mathrm{Sub}}=200\,^\circ \hbox {C}$, and $P=250$ W, while the base pressure inside the sputtering chamber is in the low $\sim 10^{-8}$ mbar range. Typical deposition rates are 0.11 nm $\hbox {s}^{-1}$ monitored by a quartz crystal monitor previously calibrated by measuring, with a 3D optical profilometer, the thickness of a deliberately deposited film.

XRD measurements are performed in grazing incident configuration by using a Rigaku Smartlab diffractometer. The primary arm is equipped with a double-bounce channel cut Ge(220) monochromator and an incident slit of 0.1 mm, which provide a monochromatic CuK$$\alpha 1$$$$(\lambda = 1.5406\, {\AA })$$ radiation. X-ray diffraction data reported in Supplementary Information are acquired by a Bruker D2 diffractometer in a specular Bragg-Brentano geometry. The system operates with a source of CuK$$\alpha$$$$(\lambda = 1.54184 \, {\AA })$$ and CuK$$\beta$$$$(\lambda = 1.39222 \, {\AA })$$ radiation at 30 kV and 10 mA.

The resistivity of the films is measured using a 4-probe van der Pauw configuration with Ag contacts realized at the four corners of unpatterned films. The high-resitivity value of the Si substrate, $\rho = 1.3 \, \Omega \,\hbox {cm}$ at room temperature, ensures that no role is played by the substrate in the measurement of the electron transport properties of the films.

The magnetic characterization is performed using a vibrating sample magnetometer. In all the reported measurements the magnetic field is applied parallel to the sample surface.

The noise characterization is carried out in a closed-cycle refrigerator at temperatures between 8 and 300 K with a temperature stabilization of $\pm \, 0.1$ K. Low-noise DC and AC electronic bias and readout are used^53,54^. A Signal Recovery 5113 amplified the AC signal, which is acquired by a dynamic signal analyzer type HP35670A controlled with LabVIEW software. The peaks visible in the voltage-noise spectra are due to external spurious sources and, therefore, are not considered in the analysis, being the relevant information in the background curves. The external magnetic field, used for noise measurements, is generated with a dipole electromagnet, type 3470 from GMW Associates. The maximum value, obtained with an operating current of 3.5 A, is 1500 Gauss.

## Supplementary information

Supplementary Information.
