# Supplementary material for: Magnetotransport and magnetic properties of amorphous NdNi5 thin films
Source: Sci Rep. 2020 Aug 13;10:13693. doi: 10.1038/s41598-020-70646-2 (PMC7426968; doi:10.1038/s41598-020-70646-2)
Supplement: Supplementary file 1 — Supplementary Information. [file 41598_2020_70646_MOESM1_ESM.pdf]

Supplementary Information for "Magnetotransport and magnetic properties of amorphous $NdNi_5$ thin films"

Carla Cirillo[1,2], Carlo Barone[2,1,3], Harry Bradshaw[4], Francesca Urban[2,1,3], Angelo Di Bernardo[5], Costantino Mauro[2], Jason W. A. Robinson[4], Sergio Pagano[2,1,3] & Carmine Attanasio[2,1,*]

[1]*CNR-SPIN, c/o Università degli Studi di Salerno, I-84084, Fisciano (Sa), Italy*

[2]*Dipartimento di Fisica "E. R. Caianiello", Università degli Studi di Salerno, I-84084, Fisciano (Sa), Italy*

[3]*INFN Gruppo Collegato di Salerno, c/o Università degli Studi di Salerno, I-84084, Fisciano (Sa), Italy*

[4]*Department of Materials Science & Metallurgy, 27 Charles Babbage Road, University of Cambridge, CB3 0FS, United Kingdom*

[5]*Universität Konstanz, Fachbereich Physik, Universitätsstraße 10, 78464 Konstanz, Germany*

[*]*cattanasio@unisa.it*

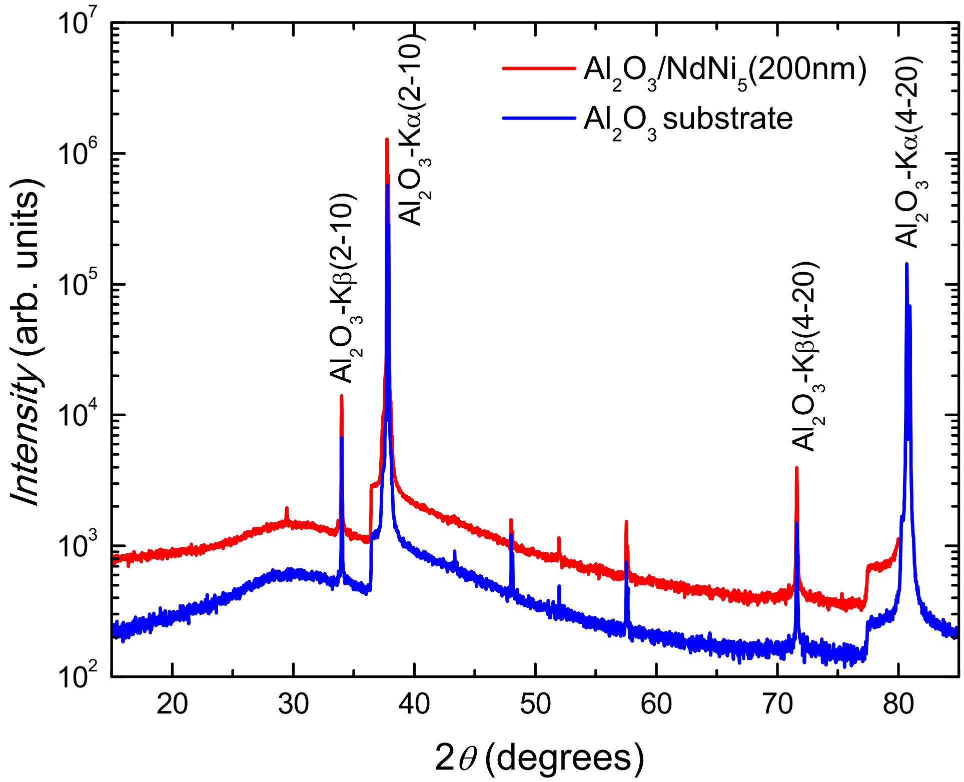

Fig. S1. **Structural characterization.** High-angle X-ray diffraction data from a 200-nm-thick $NdNi_5$ film deposited on $Al_2O_3(1120)$ (red curve). The blue curve is the trace from the bare substrate.
